# Longitudinal changes in the peripapillary retinal nerve fiber layer thickness in the fellow eyes of unilateral retinal vein occlusion

**DOI:** 10.1038/s41598-020-64484-5

**Published:** 2020-05-07

**Authors:** Yong-Il Shin, Hyung-Bin Lim, Hyungmoon Koo, Woo-Hyuk Lee, Jung-Yeul Kim

**Affiliations:** Department of Ophthalmology, Chungnam National University College of Medicine, Daejeon, Republic of Korea

**Keywords:** Eye diseases, Outcomes research

## Abstract

To analyze longitudinal changes in peripapillary retinal nerve fiber layer (pRNFL) thicknesses over time in the fellow eyes of patients with unilateral retinal vein occlusion (RVO). A total of 47 patients with unilateral RVO and 47 healthy controls were enrolled. The mean and sectoral pRNFL thicknesses were measured using spectral domain-optical coherence tomography at 1 year intervals, and followed for 3 years. Linear mixed models were performed to calculate and compare the reduction rates of pRNFL thicknesses over time. The mean pRNFL thickness decreased significantly during the 3-year follow-up, with a significant decrease over time in both groups. The reduction rate in mean pRNFL thicknesses was −0.41 μm/year in the control group and −0.68 μm/year in the fellow eyes of RVO group, and the decrease was significantly higher in the fellow eyes of RVO group than in the control group (*p* < 0.001). Using a multivariate linear mixed model, age (estimate: −0.41, *p* = 0.011) and hypertension (HTN) (estimate: −6.51, *p* = 0.014) were significantly associated with the reduction in mean pRNFL thicknesses in fellow eyes of RVO group. The fellow eyes of RVO patients showed a greater reduction in pRNFL thickness over time than normal controls. Age and HTN should be considered as factors to decrease the pRNFL thickness over time in fellow eyes of RVO group.

## Introduction

Retinal vein occlusion (RVO) is a common cause of retinal vascular disease. Branch retinal vein occlusion (BRVO) is 4–6 times more prevalent than central retinal vein occlusion (CRVO). Various systemic diseases such as hypertension (HTN), diabetes mellitus (DM), hyperlipidemia, and arteriosclerosis are considered risk factors for RVO^1–5^, so it is also important to detect and treat these systemic diseases associated with life-threatening cerebrovascular and cardiovascular diseases.

The Beijing Eye Study reported that the 10-year incidence of BRVO is 1.6%, and the incidence of CRVO was 0.3%^6^. The Beaver Dam Study estimated that the 15-year cumulative incidences of BRVO and CRVO were 1.8% and 0.5%, respectively^7^. At baseline, 5–6% of eyes had bilateral BRVO^8^, and the cumulative probability of developing a second episode of RVO in fellow eye was 7.7% within 2 years and 11.9% within 4 years^9^. Therefore, we should always consider the possibility of disease in the fellow eyes of patients with RVO.

Optical coherence tomography (OCT), developed in 1991 by Huang *et al*.^10^, is now widely used by ophthalmologists. OCT has undergone many advances in technology, with improved resolution and speed. OCT can has the advantage that it is possible to observe the change of retinal cross-section according to disease progression and treatment in high resolution. In addition, the measurement show good reproducibility and repeatability, so objective and quantitative measurements can be conducted in a short time. OCT is also widely used to quantitatively measure the thickness of the peripapillary retinal nerve fiber layer (pRNFL) during the diagnosis and treatment of glaucoma.

Several studies have reported that high intraocular pressure (IOP) and/or glaucoma were associated with the development of RVO^11–13^. Previous studies of structural changes in the fellow eyes of unilateral RVO patients have shown that the pRNFL is thinner compared to normal eyes, which suggests the possibility that RVO and glaucoma share common systemic risk factors^14^. In addition, the possibility of vascular dysfunction was reported by showing decreased perfusion of optic nerve head (ONH) measured by OCTA^15^. However, no studies have investigated longitudinal changes in pRNFL of the fellow eyes of patients with unilateral RVO. Therefore, in this study, we characterized longitudinal changes in the rate of pRNFL loss over time during a 3-year period in the fellow eyes of patients with unilateral RVO.

## Methods

### Participants

This prospective, longitudinal, observational study was approved by the Institutional Review Board of Chungnam National University Hospital, Daejeon, Republic of Korea. The study adhered to the tenets of the Declaration of Helsinki. Patients with unilateral RVO who visited our retina clinic were enrolled between January 2014 and July 2016, and the last examination was performed in August 2019. Written informed consent was obtained from each participant. The sample size was calculated using G*Power version 3.1.9.2 sample size package (http://www.gpower.hhu.de/); α = 0.05, power = 95%, effect size = 0.188 (calculate from our previous study^16^). Considering the expectable dropout, we decided to enroll at least forty participants in each group.

A retinal specialists (JYK) diagnosed unilateral RVO via dilated fundus examinations, fundus photography, and fluorescein angiography. The healthy fellow eyes of unilateral RVO patients who exhibited a best-corrected visual acuity (BCVA) of 20/25 or better were enrolled. Among the subjects who visited our clinic for various reasons (health screening checkup, work-up for ocular disease, and so forth), those who met inclusion and exclusion criteria were recruited as controls. The exclusion criteria for fellow eyes and controls were as follows: a history of retinal or optic nerve disease such as glaucoma; an IOP ≥ 21 mmHg at baseline and during the follow-up period; glaucomatous optic disc (increased cup/disc ratio, neuroretinal rim loss, disc hemorrhage, etc^17^); intraocular surgery; high myopia (spherical equivalent [SE] > −6 diopters or axial length [AL] ≥ 26.0 mm); and significant media opacity. Normal subjects (control group) also had no history of diabetes and hypertension.

All patients underwent a comprehensive ophthalmic examination, including measurement of the BCVA using a Snellen chart, slit-lamp examination, dilated fundus examination, IOP, SE, AL measurement using an IOL Master version 5.0 (Carl Zeiss, Jena, Germany), and spectral domain-optical coherence tomography (SD-OCT). After the initial visit, the examinations were performed at 1 year intervals between examinations for 3 years.

### Optical coherence tomography measurements

SD-OCT imaging was performed using a Cirrus HD-OCT version 10.0 (Carl Zeiss Meditec, Dublin, CA, USA), by a single experienced examiner. The pRNFL thickness was measured using an optic disc cube (200 × 200) scan mode. This mode scanned a 6 × 6 mm area and measured the pRNFL thickness of a 3.46 diameter circle from the optic disc center. The average and four quadrant sectors (superior, nasal, inferior, and temporal) thicknesses were used for pRNFL analyses. We excluded the following: a signal strength <7, a segmentation error, a motion artifact, or poor centration.

### Statistical analysis

All statistical analyses were performed using SPSS statistical software for Windows version 22.0 (SPSS, Chicago, IL, USA). For statistical analyses, BCVA values were transformed into the logarithm of the minimum angle of resolution (LogMAR) values. The independent *t-*test and chi-square test were used to compare clinical factors, and compare ocular parameters between fellow eyes of RVO patients and normal controls.

Linear mixed models were used to calculate and compare the reduction rates of pRNFL thicknesses over time between fellow eyes of RVO patients and normal controls. The pRNFL thickness was fitted with linear mixed models using age, sex, BCVA, IOP, AL, SE, baseline average pRNFL thickness, HTN, DM, and the interactions between the group and follow-up durations as fixed effects. A random intercept was included at eye levels. Univariate and multivariate generalized linear mixed models were used to determine the factors associated with longitudinal changes in pRNFL thicknesses.

## Results

### Demographics and clinical characteristics

A total 58 unilateral RVO patients and 60 normal subjects were initially included in this study; 24 individuals were excluded due to loss to follow-up (n = 19), cataract surgery (n = 3), or withdraw the consent (n = 2). As a result, a total of 47 patients (17 males and 30 females) with unilateral RVO (39 BRVO and 8 CRVO) and 47 healthy controls (21 males and 26 females) were enrolled. About 70.2% of RVO patients were diagnosed with HTN and 19.1% had DM. There were no significant differences in BCVA, SE, IOP, or AL between the fellow eyes of the RVO group and the controls (Table 1). In the comparison of baseline pRNFL thicknesses, no significant difference was found between two groups except the temporal sector (p = 0.023).

**Table 1:** Demographics and clinical characteristics.

	Retinal vein occlusion (n = 47)	Control group (n = 47)	*p*-value
Age (years)	66.2 ± 7.1	65.1 ± $7$.2	0.453^†^
Sex (male/ female)	17/30	21/26	0.401^‡^
Hypertension (no. (%))	33 (70.2%)	0 (0%)	NA
Diabetes (no. (%))	9 (19.1%)	0 (0%)	NA
BCVA (logMAR)*	0.00 ± 0.08	−0.02 ± 0.07	0.251^†^
Spherical equivalent (diopters)*	0.46 ± 1.41	0.23 ± 1.22	0.393^†^
Intraocular pressure (mmHg)*	15.7 ± 2.5	16.4 ± 2.1	0.161^†^
Axial length (mm)*	23.4 ± 0.9	23.6 ± 0.7	0.144^†^
Baseline pRNFL thickiness (μm)*			
Average	95.9 ± 8.0	98.7 ± 7.4	0.083^†^
Superior	121.8 ± 14.9	122.5 ± 14.0	0.814^†^
Nasal	69.1 ± 11.2	70.3 ± 7.7	0.527^†^
Inferior	124.0 ± 14.1	129.1 ± 14.8	0.091^†^
Temporal	68.4 ± 8.4	73.0 ± 10.6	0.023^†^

BCVA = best-corrected visual acuity; logMAR = logarithm of the minimum angle of resolutions; pRNFL = peripapillary retinal nerve fiber layer.

Values are presented as mean ± SD unless otherwise indicated.

*Values are for the fellow eyes in the RVO group.

^†^By independent *t*-test.

^‡^ By chi-square test.

### Mean and sectoral pRNFL thicknesses at each visit

In fellow eyes of RVO group, the mean pRNFL thickness decreased significantly during 3-year follow-up and the reduction was significantly decreased over time using the linear mixed model (*p* < 0.001). Futhermore, the pRNFL thicknesses of all sectors (superior, nasal, inferior, and temporal) showed a significant reduction over time (all, *p* < 0.05). In control group, the mean pRNFL thickness also decreased significantly over time (*p* = 0.042). However, in sectoral analysis, only the superior and inferior sectors decreased significantly over time (Table 2).

**Table 2:** Peripapillary retinal nerve fiber layer thicknesses at each visit.

	Fellow eye of RVO	*p -* value	Control group	*p -* value
**Mean**				
Baseline	95.9 ± 8.0	**0.001**	98.7 ± 7.4	**0.042**
First year	95.1 ± 8.5		98.1 ± 7.7	
Second year	94.0 ± 8.5		97.8 ± 7.5	
Third year	94.0 ± 9.0		97.0 ± 8.1	
**Superior**				
Baseline	121.8 ± 14.9	**0.022**	122.5 ± 14.0	**0.043**
First year	120.7 ± 14.5		121.7 ± 13.9	
Second year	119.1 ± 13.7		120.8 ± 12.1	
Third year	119.0 ± 15.0		120.0 ± 12.0	
**Nasal**				
Baseline	69.1 ± 11.2	**0.004**	70.3 ± 7.7	0.863
First year	68.3 ± 11.3		70.3 ± 9.0	
Second year	67.5 ± 9.7		70.3 ± 9.6	
Third year	67.5 ± 11.0		70.1 ± 9.5	
**Inferior**				
Baseline	124.0 ± 14.1	**<0.001**	129.1 ± 14.8	**0.033**
First year	123.4 ± 16.0		127.7 ± 13.9	
Second year	121.6 ± 15.4		127.8 ± 13.4	
Third year	121.4 ± 15.2		126.5 ± 14.9	
**Temporal**				
Baseline	68.4 ± 8.4	**0.026**	73.0 ± 10.6	0.343
First year	68.1 ± 8.9		72.8 ± 11.0	
Second year	67.4 ± 8.4		72.3 ± 11.6	
Third year	66.8 ± 7.9		71.6 ± 12.1	

Values are presented as mean ± SD.

### Reduction rates of pRNFL thickness and factors associated with mean pRNFL reduction

**Figure 1:**
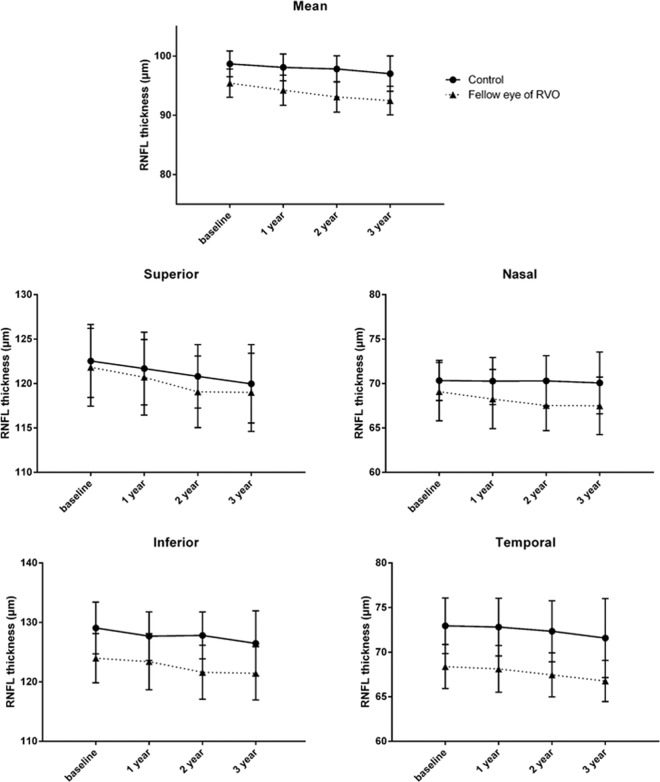
The mean and four sectorial thicknesses of peripapillary retinal nerve fiber layer of each group’s with the mean with the 95% confidential interval at each visit. The solid line represents the fellow eyes of retinal vein occlusion group, and the dotted line represents the control group. There were significant differences between the two groups in all areas except temporal sector.

The reduction rates of mean pRNFL thickness were −0.68 and −0.41 µm/year in the fellow eyes and controls, respectively. Reduction rates were significantly associated with the interaction between groups and follow-up duration using linear mixed model (*p* < 0.001). This means that there was a significant difference in the reduction rate over time between the two groups. In sectoral analysis, there were significant differences between the two groups in the superior, nasal, and inferior, but not temporal sectors (Fig. 1) (Table 3).

**Table 3:** Rate of change in peripapillary retinal nerve fiber layer thickness.

	Fellow eye of RVO	Control group	p - value *
Mean	−0.68 (−1.05 to −0.30)	−0.41 (−0.80 to −0.02)	**<0.001**
Superior	−1.03 (−1.91 to −0.16)	−0.78 (−1.53 to −0.03)	**0.009**
Nasal	−0.67 (−1.12 to −0.22)	−0.04 (−0.50 to 0.47)	**0.011**
Inferior	−1.08 (−1.67 to −0.49)	−0.77 (−1.47 to −0.07)	**<0.001**
Temporal	−0.54 (−1.02 to −0.07)	−0.24 (−0.75 to 0.27)	0.053

*p- value for interaction between group and duration in linear mixed models. Values are presented as μm/year (95% confidence interval).

In multivariate linear mixed model analyses, age (estimate: −0.41, *p* = 0.011) and HTN (estimate: −6.51, *p* = 0.014) were associated with longitudinal changes in thickness in fellow eyes of the RVO group (Table 4).

**Table 4:** Univariate and multivariate linear mixed-effect model determination of factors associated with changes of peripapillary retinal nerve fiber layer thickness in the fellow eye of retinal vein occlusion.

	Univariate		Multivariate	
	Estimate (μm/year, 95% CI)	*p*-value	Estimate (μm/year, 95% CI)	*p*-value
Age	−0.41 (−0.75, −0.06)	0.022	−0.41 (−0.73, −0.10)	0.011
Sex	−1.37 (−7.10, 4.36)	0.630		
BCVA	−23.71 (−61.45, 14.02)	0.210		
Intraocular pressure	−0.27 (−1.37, 0.84)	0.629		
Axial length	−1.39 (−4.60, 1.81)	0.383		
Spherical equivalent	−0.09 (−1.91, 1.74)	0.924		
Hypertension	−6.48 (−12.07, 0.88)	0.025	−6.51 (−11.64, −1.38)	0.014
Diabetes	−0.49 (−1.31, −0.33)	0.234		

CI = confidence interval; BCVA = best-corrected visual acuity.

To confirm the effect of hypertension, the fellow eyes of the RVO group were divided into two groups according to HTN status (HTN: 33 eyes, no HTN: 14 eyes), and the reduction rate of the average pRNFL thickness was compared. As a result, the reduction rate in the HTN group was −0.89 µm/year (95% CI: −1.22, −0.56; p < 0.001), which was significantly different from the control group (p < 0.001), and no HTN group (−0.14 µm/year; 95% CI: −0.62, 0.34; p = 0.550) showed no difference from the control group (p = 0.357).

## Discussion

The purpose of the present study was to investigate changes in peripapillary RNFL thickness over time in the fellow eyes of unilateral RVO patients. The RNFL thickness in the fellow eye of unilateral RVO patients was thinner than that of controls and significantly decreased over time. In addition, the rate of reduction was greater than that of the controls. Factors associated with this reduction in RNFL were age, and HTN.

The RNFL consists of axons of the ganglion cell layer of the retina that form nerve fiber bundles and converge upon the ONH. The fibers originating from the macula run horizontally toward the ONH and form papillomacular bundles. The fibers originating from the temporal region of the macular run in an arcuate shape to avoid the horizontal raphe, toward the ONH. Early detection of an RNFL defect is important because glaucoma patients are sensitive to changes in RNFL before vision loss or before a visual field defect occurs^18^. The RNFL thickness is measured automatically in the OCT as a highly reflective layer at the top of the vitreoretinal interface. Typically, the thickness of the superior, nasal, inferior, and temporal quadrants and the 12 o’clock hour around the ONH are shown, and the distribution of the RNFL is displayed in a map. In addition, localized and diffuse RNFL defects can be detected more easily by displaying the color of the abnormal probability by comparing with normative data.

The mean number of nerve fibers in the optic nerve head is about 1 million. Histological studies have reported that the rate of loss of ganglion cell axons increase with age^19^. Histomorphometry studies has shown that there is significant loss of 5426 optic nerves per year of age^20^. Several cross-sectional studies that have used OCT have also reported a decrease in RNFL with age, with an annual reduction rate of at least −0.16 µm/year and as high as −0.44 µm/year in normal individuals^21–24^. In a prospective, longitudinal study of RNFL reduction in normal subjects, the reduction rate was −0.52 µm/year when 35 patients were observed an average of 30 months at 4-month intervals^25^. In our study, normal controls showed that the mean RNFL thickness reduction rate was −0.41 μm/year and the 95% of the mean estimation ranged between −0.80 and −0.02 μm/year. Similar to the previous study^21–24^, the reduction rate was higher in the superior and inferior quadrants.

Previously, RVO has been known to have several associations with glaucoma. If the two diseases share a common risk factor, then unilateral RVO patients may also show changes in RNFL thickness in the fellow eye^14^. In the fellow eyes of unilateral RVO patients, RNFL thickness showed sector thinning at 7, 10, and 11 o’clock compared to normal control. Since the location is the most frequent site of glaucomatous change, the possibility of association with glaucoma was explained^14^. Shin *et al*.^15^ reported a decrease in ONH microvascular perfusion in fellow eyes of unilateral RVO patients, as well as an association with vascular dysfunction, which is a mechanisms of glaucoma. Therefore, we conducted a prospective longitudinal study to determine whether there was a difference in the RNFL thickness reduction rates between the two groups, as well as to determine whether any factors were associated with these reductions.

The mean pRNFL thickness decreased significantly during 3-year follow-up, and the reduction was significantly decreased over time in the fellow eyes of unilateral RVO patients. Fellow eyes of unilateral patients showed that the average RNFL thickness reduction rate was −0.68 μm/year and the 95% of the mean estimation ranged between −1.05 and −0.30 μm/year. In addition, there were significant differences in the superior, nasal, and inferior sectors between the two groups. Only the temporal sector showed no statistical difference, but the *p*-value was 0.056. DM and HTN are well known risk factors for RVO. In a previous 4-year follow-up longitudinal study of the reduction rates of pRNFL related to systemic diseases, the reported rates were −0.92 μm/year in the non-diabetic retinopathy group (DR) and −1.16 μm/year in the non-proliferative DR group^26^, and −0.99 μm/year in a HTN patients^16^. Because about 70.2% of patients in our study were treated with HTN and 19.1% were treated for DM, the reduction rate of pRNFL thickness in the fellow eye of unilateral RVO patients may be significantly higher than in normal controls in association with these systemic disease.

Our study showed that age and HTN were significantly associated with reduction rate of RNFL thickness. Kim *et al*.^14^ studied the fellow eyes of RVO patients, and reported a statistically significant reduction in the subgroup analysis of only patients >60 years of age, compared to controls, which may have been associated with aging. In particular, patients with high myopia, 50–59 years of age, and 40–49 years of age showed a significant reduction over time compared with the normal controls. The rate change in pRNFL of normal controls was −0.19 between 20–29 years of age and −0.63 μm/year at 50–59 years of age, which was greater in older subjects^27^. It is also possible that the prevalence of cardiovascular disease increased with age, and ischemic effects on the retina and optic nerve related to systemic disease may have affected the decrease in RNFL thicknesses. In our study, DM did not show statistical significance association, which was likely due to the relatively small number of patients receiving DM treatment, as well as the short duration of DM.

Our study had some limitations. We excluded patients with an IOP > 21 mmHg, with glaucomatous optic disc, or with obvious RNFL defects during the enrollment and follow-up periods, but we were unable to completely rule out patients with pre-perimetric glaucoma because visual field examinations were not performed and patient’s data was not reviewed by a glaucoma specialist. Further study including visual field examinations may therefore be necessary. In addition, more detailed prospective longitudinal studies that include factors such as the types of HTN medications taken by patients and the presence of blood pressure (BP) fluctuations using 24 hour BP monitoring are needed to determine the effects of HTN on the fellow eyes of RVO patients. However, we prospectively observed changes over a relatively long period of 3 years, which is an advantage as the first study to help understand the structural changes in the longitudinal changes in RNFL reduction in the fellow eye of RVO patients.

In conclusion, the rate of pRNFL thinning in the fellow eyes of unilateral RVO patients was greater than that in normal eyes and was related to age and HTN. Ophthalmologists should be careful to interpret the reduction of RNFL thickness when treating these patients.

## Data Availability

Data supporting the findings of the current study are available from the corresponding author on reasonable request.
